# Threshold for defining PSMA-positivity prior to ^177^Lu-PSMA therapy: a comparison of [^68^Ga]Ga-PSMA-11 and [^18^F]F-DCFPyL in metastatic prostate cancer

**DOI:** 10.1186/s13550-023-01033-x

**Published:** 2023-09-20

**Authors:** Jan Heilinger, Jasmin Weindler, Katrin Sabine Roth, Philipp Krapf, Klaus Schomäcker, Markus Dietlein, Alexander Drzezga, Carsten Kobe

**Affiliations:** 1 Department of Nuclear Medicine, Faculty of Medicine and University Hospital Cologne, University of Cologne, Kerpener Straße 62, 50937 Cologne, Germany; 2 Institute of Neuroscience and Medicine, Nuclear Chemistry (INM-5), Forschungszentrum Jülich GmbH, Wilhelm-Johnen-Straße, 52428 Jülich, Germany

**Keywords:** Prostate cancer, PSMA, PET, Radionuclide therapy, Theranostics, Quantification, SUV

## Abstract

**Background:**

In 2022, the American Food and Drug Administration and the European Medicines Agency approved [^177^Lu]Lu-PSMA-617 (PLUVICTO™, Novartis AG, Basel, Switzerland) for radionuclide therapy with prostate-specific membrane antigen (PSMA) ligands in metastatic prostate cancer. Theranostics require appropriate patients to be identified by positron emission tomography (PET) prior to radionuclide therapy, usually employing [^68^Ga]Ga-PSMA-11. Alternatively, several ^18^F-labelled PSMA-PET tracers are available and may increasingly replace ^68^Ga-labelled compounds, with respect to their image quality, availability and other practical advantages. However, alternative tracers may differ in uptake behaviour, and their comparability with regard to patient selection for [^177^Lu]Lu-PSMA therapy has not yet been established. Here, we analysed whether tumour-to-background ratios determined by PET using the ^18^F-labelled PSMA-specific radiopharmaceutical [^18^F]F-DCFPyL were comparable to those determined by PET using [^68^Ga]Ga-PSMA-11.

**Results:**

No differences could be observed between [^68^Ga]Ga-PSMA-11-PET and [^18^F]F-DCFPyL-PET regarding tumour-to-liver ratios or tumour-to-mediastinum ratios (e. g. tumour-to-liver ratios using maximum SUV of the tumour lesion for ultra-high definition reconstructed PET images with a median of 2.5 (0.6–9.0) on [^68^Ga]Ga-PSMA-11-PET vs. 2,0 (0.6–11.4) on [^18^F]F-DCFPyL-PET). However, significant differences were observed in terms of contrast-to-noise ratios, thereby demonstrating the better image quality obtained with [^18^F]F-DCFPyL-PET.

**Conclusions:**

Our data showed that [^18^F]F-DCFPyl-PET and [^68^Ga]Ga-PSMA-11-PET provide comparable tumour-to-liver and tumour-to-mediastinum ratios. Therefore, a tumour uptake of [^18^F]F-DCFPyL above the liver background, like using [^68^Ga]Ga-PSMA-11, can be considered as equally suitable for defining PSMA-positivity by a semiquantitative assessment based on the liver background, e. g. prior to radioligand therapy with ^177^Lu-labelled PSMA ligands. In addition, our data suggest a tending advantage of [^18^F]F-DCFPyL in terms of lesion detectability.

## Background

Prostate cancer is a major public health concern, as it is the second most common malignancy in adult males worldwide [1]. Despite multimodal treatments that delay disease progression, advanced metastatic prostate cancer remains fatal [2, 3].

Nuclear medicine is becoming increasingly important in the treatment of prostate cancer, using overexpression of the prostate-specific membrane antigen (PSMA) on the surface of prostate cancer cells to internalise intravenously administered, radiolabelled PSMA ligands for diagnostic and therapeutic purposes [4, 5, 6, 7, 8, 9]. Such PSMA ligands can be labelled with positron emitters (e.g. gallium-68, fluorine-18), allowing diagnostic imaging by positron emission tomography (PET), or with beta emitters (e.g. lutetium-177) to perform radioligand therapy [4, 5].

Radioligand therapy with ^177^Lu-labelled PSMA ligands has been used successfully for several years as part of individualised treatment plans for patients with prostate cancer [10]. In 2022, based on the PSMA-VISION trial, [^177^Lu]Lu-PSMA-617 (PLUVICTO™, Novartis AG, Basel, Switzerland) became the first PSMA-directed radiopharmaceutical to be formally approved by the American Food and Drug Administration and the European Medicines Agency for the treatment of patients with PSMA-positive metastatic castration-resistant prostate cancer who had previously been treated with androgen receptor pathway-inhibiting drugs and taxane-based chemotherapy [11, 12].

Although PSMA overexpression is a common finding in patients with prostate cancer, there is a considerable intra- and interpatient heterogeneity [13, 14]. Appropriate patients should therefore be identified by PSMA-PET prior to radioligand therapy according to the principles of theranostics [3, 4, 10]. In the PSMA-VISION trial, patients were required to have PSMA-positive tumour lesions, defined as PSMA expression above the liver background on PET using [^68^Ga]Ga-PSMA-11 [3]. In order to be able to offer radioligand therapy based on PET diagnostics with an alternative ^18^F-labelled PSMA tracer, we aimed to address the question whether employing the ^18^F-labelled PSMA-specific radiopharmaceutical [^18^F]F-DCFPyL would result in comparable patient classification to established [^68^Ga]Ga-PSMA-11-PET [15]. For this purpose, we compared PET scans of patients who had undergone both PET with [^68^Ga]Ga-PSMA-11 and [^18^F]F-DCFPyL within a short time interval as part of their clinical workup. We measured uptake levels of metastases and background regions (liver, mediastinum) in order to calculate and compare tumour-to-background ratios. As tumour expression of PSMA above the hepatic background was the main criterion for PSMA-positivity in the PSMA-VISION trial, we paid particular attention to differences in the tumour-to-liver ratio between PET images using [^68^Ga]Ga-PSMA-11 and [^18^F]F-DCFPyL [3].

## Methods

### Patients and PET

Within this retrospective study, we analysed a total of 11 patients who had undergone both a PET scan with [^68^Ga]Ga-PSMA-11 and a PET scan with [^18^F]F-DCFPyL in short succession as part of their clinical workup between July 2014 and December 2016. All patients had a biochemical recurrence of their prostate cancer and had presented for restaging in order to plan their further treatment. The selection of patients for an additional PET scan using [^18^F]F-DCFPyL was based on the assumption that adding further diagnostic information would significantly improve the treatment decision in each individual case. The same group of patients has already been the subject of another publication focusing on different aspects [16].

Every patient underwent the following procedure for each tracer using a Biograph 16 TruePoint system (Siemens Medical Solutions, Erlangen, Germany) in six patients and a Biograph mCT 128 Flow-Edge system (Siemens Medical Solutions, Erlangen, Germany) in five patients. First, native non-diagnostic computed tomography (CT) was performed for attenuation correction from skull to the mid-thigh. Next, the PET scan was carried out covering that same region. To ensure comparability between different PET/CT systems, reconstruction was performed via an ordered subset expectation maximisation (OSEM) algorithm (4 iterations and 14 subsets) and a HD (high definition) algorithm (3 iterations and 21 subsets), both followed by an intrinsic 5-mm Gaussian filter in all directions for the Siemens Biograph 16 TruePoint system. Reconstruction via an OSEM algorithm (4 iterations and 12 subsets), followed by an intrinsic 5-mm Gaussian filter in all directions, and a UHD (ultra-high definition) algorithm (3 iterations and 21 subsets), followed by an intrinsic 2-mm Gaussian filter in all directions, were performed for the Siemens Biograph mCT 128 Flow-Edge system.

### Quantitative analysis

The PET images were quantitatively analysed using the software syngo.via (Siemens Healthineers, Erlangen, Germany). All subsequent evaluations have been performed separately using both OSEM and HD/UHD reconstructed PET images.

We compared PET scans with [^68^Ga]Ga-PSMA-11 and [^18^F]F-DCFPyL for each patient. First, tumour lesions that were reliably recognisable and well delineated on both scans were identified. Separate evaluations were then performed for each of these lesions, determining standardised uptake values corrected for body weight (SUV). For this, tumour lesions were segmented using 41% of their maximum SUV as a cut-off [17]. Maximum and mean SUV of the tumour lesions (SUV_maxT_ and SUV_meanT_) were measured in the resulting volumes.

In addition, general background levels were measured for each patient in both PET scans. This included hepatic and mediastinal backgrounds. The mean SUV in the liver (SUV_meanL_) was determined by placing a 3.0-cm-diameter spherical volume of interest (VOI) in the inferior right part of the normal liver [18]. The mean SUV in the mediastinum (SUV_meanM_) was determined by placing a spherical VOI with a diameter of 2.0 cm in the thoracic aorta [18].

Moreover, the local background level in the tissue surrounding the tumour was measured for each tumour lesion in both PET scans. For this purpose, mean SUV and its standard deviation in the local background (SUV_meanN_ and SD_N_) were determined by placing a 3.0-cm-diameter spherical VOI in the tissue around the tumour [19].

Ratios of the maximum SUV in the tumour lesion to the mean SUVs in the liver and mediastinum, respectively, as well as ratios of the mean SUV in the tumour lesion to the mean SUVs in the liver and mediastinum were calculated for both PET scans (tumour-to-liver ratios, tumour-to-mediastinum ratios: SUV_maxT_/SUV_meanL_, SUV_meanT_/SUV_meanL_, SUV_maxT_/SUV_meanM_, SUV_meanT_/SUV_meanM_).

In addition, contrast-to-noise ratios were calculated for each lesion in both PET scans, defined as follows: contrast-to-noise ratio = [SUV_meanT_—SUV_meanN_]/SD_N_ [19].

### Statistical analysis

Statistical analyses were performed with the software SPSS statistics 29.0.0.0 (IBM, Armonk, NY, USA). All subsequent analyses were performed separately, using both data from OSEM and HD/UHD reconstructed PET images.

Basic descriptive statistics were performed for patient characteristics, tumour-to-liver ratios, tumour-to-mediastinum ratios and contrast-to-noise ratios.

Tumour-to-liver ratios, tumour-to-mediastinum ratios and contrast-to-noise ratios were compared between [^68^Ga]Ga-PSMA-11-PET and [^18^F]F-DCFPyL-PET using the Wilcoxon matched-pair signed-rank [2 samples] test. A p-value of p < 0.05 was considered statistically significant.

Results for tumour-to-liver ratios, tumour-to-mediastinum ratios and contrast-to-noise ratios were graphically visualised using boxplots.

## Results

### Patients

The median age of the patients was 68 years (53–86 years), the median body weight was 88 kg (62–124 kg), and the median prostate-specific antigen (PSA) level in the patients’ blood was 3.0 ng/ml (1.2–50.0 ng/ml). The median time interval between [^68^Ga]Ga-PSMA-11-PET and [^18^F]F-DCFPyL-PET was 13 days (6–41 days). The median applied activity for [^68^Ga]Ga-PSMA-11-PET was 139 MBq (64–187 MBq) compared to 350 MBq (240–411 MBq) for [^18^F]F-DCFPyL-PET. Image acquisition started at a median of 65 min (49–122 min) after application of [^68^Ga]Ga-PSMA-11 and a median of 113 min (77–128 min) after application of [^18^F]F-DCFPyL. A total of 24 concordant tumour lesions were identified in both PET scans including 15 lymph node metastases, six local recurrences and three bone metastases. Patient characteristics are shown in Table 1.

**Table 1:** Patient characteristics and PET parameters

Patient no.	Age (years)	Body weight (kg)	PSA (ng/l)	Activity (MBq)	Time to image acquisition (min)	PET-scanner
1	53	85	4.10			
^*68*^*Ga*				177	61	Siemens Biograph 16
^*18*^*F*				350	175	Siemens Biograph 16
2	86	74	2.10			
^*68*^*Ga*				139	109	Siemens Biograph 16
^*18*^*F*				347	170	Siemens Biograph 16
3	74	100	4.70			
^*68*^*Ga*				138	122	Siemens Biograph 16
^*18*^*F*				364	93	Siemens Biograph 16
4	82	91	50.00			
^*68*^*Ga*				139	65	Siemens Biograph 16
^*18*^*F*				382	112	Siemens Biograph 16
5	68	90	n. a			
^*68*^*Ga*				104	49	Siemens Biograph 16
^*18*^*F*				411	126	Siemens Biograph 16
6	68	94	1.30			
^*68*^*Ga*				139	129	Siemens Biograph 16
^*18*^*F*				349	128	Siemens Biograph 16
7	68	88	1.20			
^*68*^*Ga*				110	74	Siemens Biograph mCT 128 Edge
^*18*^*F*				240	123	Siemens Biograph mCT 128 Edge
8	60	124	2.04			
^*68*^*Ga*				95	49	Siemens Biograph mCT 128 Edge
^*18*^*F*				280	94	Siemens Biograph mCT 128 Edge
9	74	76	3.87			
^*68*^*Ga*				64	57	Siemens Biograph mCT 128 Edge
^*18*^*F*				360	107	Siemens Biograph mCT 128 Edge
10	54	62	10.00			
^*68*^*Ga*				187	64	Siemens Biograph mCT 128 Edge
^*18*^*F*				297	117	Siemens Biograph mCT 128 Edge
11	76	75	1.48			
^*68*^*Ga*				160	67	Siemens Biograph mCT 128 Edge
^*18*^*F*				363	49	Siemens Biograph mCT 128 Edge

PET positron emission tomography, PSA prostate-specific antigen in blood test, ^68^Ga [^68^Ga]Ga-PSMA-11, ^18^F [^18^F]F-DCFPyL, n. a. not available

### *Tumour-to-background ratios of [*^*68*^*Ga]Ga-PSMA-11 and [*^*18*^*F]F-DCFPyL are comparable*

We calculated various tumour-to-background ratios in OSEM and HD/UHD reconstructed [^68^Ga]Ga-PSMA-11-PET and [^18^F]F-DCFPyL-PET, as shown in Tables 2 and 3.

**Table 2:** Tumour-to-background ratios including contrast-to-noise ratios from OSEM-PET

Lesion no.	SUV_maxT_/SUV_meanL_		SUV_meanT_/SUV_meanL_		SUV_maxT_/SUV_meanM_		SUV_meanT_/SUV_meanM_		CNR	
	^*68*^*Ga*	^*18*^*F*	^*68*^*Ga*	^*18*^*F*	^*68*^*Ga*	^*18*^*F*	^*68*^*Ga*	^*18*^*F*	^*68*^*Ga*	^*18*^*F*
1	0.79	0.64	0.65	0.42	2.49	2.21	2.04	1.48	7.37	6.61
2	1.06	1.00	0.59	0.55	3.34	3.49	1.84	1.91	7.22	6.68
3	2.32	1.56	1.35	0.98	11.94	11.23	6.94	7.10	16.18	11.29
4	1.75	1.41	1.02	0.90	9.02	7.13	5.27	4.55	21.18	27.85
5	0.92	0.77	0.53	0.52	4.73	3.89	2.74	2.61	12.05	21.11
6	8.76	8.50	4.79	4.39	30.39	26.70	16.61	13.80	24.22	28.96
7	8.70	9.91	5.64	6.23	30.17	31.12	19.55	19.57	49.29	80.03
8	5.35	6.63	3.43	4.03	18.57	20.83	11.89	12.67	29.40	51.24
9	2.68	2.66	1.61	1.71	9.28	8.36	5.58	5.38	13.02	20.83
10	2.03	3.68	1.24	2.31	7.04	11.56	4.31	7.26	9.74	28.69
11	5.40	5.29	3.06	3.23	18.72	16.60	10.61	10.15	22.94	42.64
12	1.71	2.40	1.05	1.45	5.94	7.55	3.64	4.56	8.00	17.41
13	4.10	3.74	2.63	2.52	11.38	15.93	7.31	10.76	14.21	24.49
14	1.58	0.81	0.40	0.49	5.30	4.44	1.34	2.71	3.92	11.11
15	4.15	1.74	2.31	1.04	18.00	9.98	10.04	5.94	32.86	27.54
16	1.90	2.54	1.19	1.49	5.41	10.23	3.38	5.99	9.33	33.40
17	0.63	0.73	0.36	0.43	2.63	3.12	1.49	1.83	5.00	22.00
18	0.27	0.44	0.15	0.25	1.13	1.86	0.63	1.07	2.63	14.13
19	0.54	0.57	0.38	0.33	2.25	2.44	1.57	1.42	1.75	7.40
20	0.71	0.76	0.42	0.43	2.96	3.23	1.75	1.86	2.23	11.40
21	0.76	0.72	0.42	0.39	3.15	3.07	1.74	1.67	4.03	5.52
22	1.31	1.24	0.79	0.75	5.24	5.87	3.16	3.55	11.74	27.73
23	0.66	0.40	0.39	0.22	2.65	1.88	1.56	1.05	6.24	3.33
24	1.80	0.94	0.91	0.50	6.77	1.92	3.43	1.02	21.42	23.67

SUV standardised uptake values corrected for body weight, OSEM ordered subset expectation maximisation, PET positron emission tomography, SUVmaxT/SUVmeanL Ratio of the maximum SUV in the tumour lesion to the mean SUV in the liver, SUVmeanT/SUVmeanL Ratio of the mean SUV in the tumour lesion to the mean SUV in the liver, SUVmaxT/SUVmeanM Ratio of the maximum SUV in the tumour lesion to the mean SUV in the mediastinum, SUVmeanT/SUVmeanM Ratio of the mean SUV in the tumour lesion to the mean SUV in the mediastinum, CNR contrast-to-noise ratio, ^68^Ga [^68^Ga]Ga-PSMA-11, ^18^F [^18^F]F-DCFPyL

**Table 3:** Tumour-to-background ratios including contrast-to-noise ratios from HD/UHD-PET

Lesion no	SUV_maxT_/ SUV_meanL_		SUV_meanT_/ SUV_meanL_		SUV_maxT_/ SUV_meanM_		SUV_meanT_/ SUV_meanM_		CNR	
	^*68*^*Ga*	^*18*^*F*	^*68*^*Ga*	^*18*^*F*	^*68*^*Ga*	^*18*^*F*	^*68*^*Ga*	^*18*^*F*	^*68*^*Ga*	^*18*^*F*
1	0.80	0.57	0.54	0.38	2.24	1.66	1.51	1.09	9.28	7.83
2	1.18	0.84	0.59	0.53	3.31	2.42	1.65	1.52	7.91	6.67
3	3.13	1.63	1.91	1.02	13.17	10.00	8.04	6.26	26.71	30.52
4	2.49	1.65	1.45	1.03	9.84	7.00	5.71	4.36	31.00	52.06
5	1.55	1.13	0.94	0.74	6.14	4.80	3.71	3.15	18.95	27.90
6	8.81	9.43	4.76	5.01	27.59	26.13	14.90	13.88	28.78	71.96
7	8.96	11.38	5.83	7.00	28.04	31.55	18.24	19.40	111.18	151.50
8	6.74	8.51	4.02	5.28	21.09	23.61	12.59	14.63	75.73	113.11
9	2.69	3.18	1.67	2.10	8.41	8.81	5.22	5.82	29.55	42.11
10	2.89	3.87	1.82	2.45	9.06	10.72	5.69	6.78	32.45	49.83
11	4.57	5.84	2.68	3.45	14.32	16.19	8.38	9.57	45.00	54.17
12	1.61	2.09	1.01	1.28	5.03	5.80	3.17	3.56	16.64	23.89
13	4.70	3.80	3.07	2.46	11.11	13.28	7.26	8.61	26.24	35.16
14	1.19	1.01	0.72	0.56	3.37	4.39	2.04	2.46	12.06	12.75
15	6.24	3.02	3.73	1.77	27.50	17.25	16.43	10.10	32.17	46.88
16	4.73	6.60	3.54	5.20	11.97	27.35	8.97	21.54	27.13	75.20
17	1.23	2.74	0.86	1.74	5.02	11.03	3.53	7.01	12.23	46.10
18	0.61	1.22	0.38	0.94	2.48	4.89	1.55	3.76	6.50	35.07
19	1.07	1.30	0.65	0.81	4.38	5.21	2.64	3.26	3.12	12.07
20	1.49	1.80	0.87	1.04	6.11	7.24	3.57	4.20	4.81	16.75
21	0.87	1.40	0.53	0.86	3.55	5.65	2.17	3.44	4.66	13.04
22	2.56	2.58	1.60	1.80	9.32	10.76	5.82	7.50	22.26	36.62
23	1.26	0.60	0.78	0.35	4.59	2.50	2.83	1.45	11.65	3.34
24	4.25	1.76	2.51	0.93	16.28	3.41	9.62	1.80	48.95	27.38

SUV standardised uptake values corrected for body weight, HD high definition, UHD ultra-high definition, PET positron emission tomography, SUVmaxT/SUVmeanL Ratio of the maximum SUV in the tumour lesion to the mean SUV in the liver, SUVmeanT/SUVmeanL Ratio of the mean SUV in the tumour lesion to the mean SUV in the liver, SUVmaxT/SUVmeanM Ratio of the maximum SUV in the tumour lesion to the mean SUV in the mediastinum, SUVmeanT/SUVmeanM Ratio of the mean SUV in the tumour lesion to the mean SUV in the mediastinum, CNR contrast-to-noise ratio, ^68^Ga [^68^Ga]Ga-PSMA-11, ^18^F [^18^F]F-DCFPyL

**Fig. 1:**
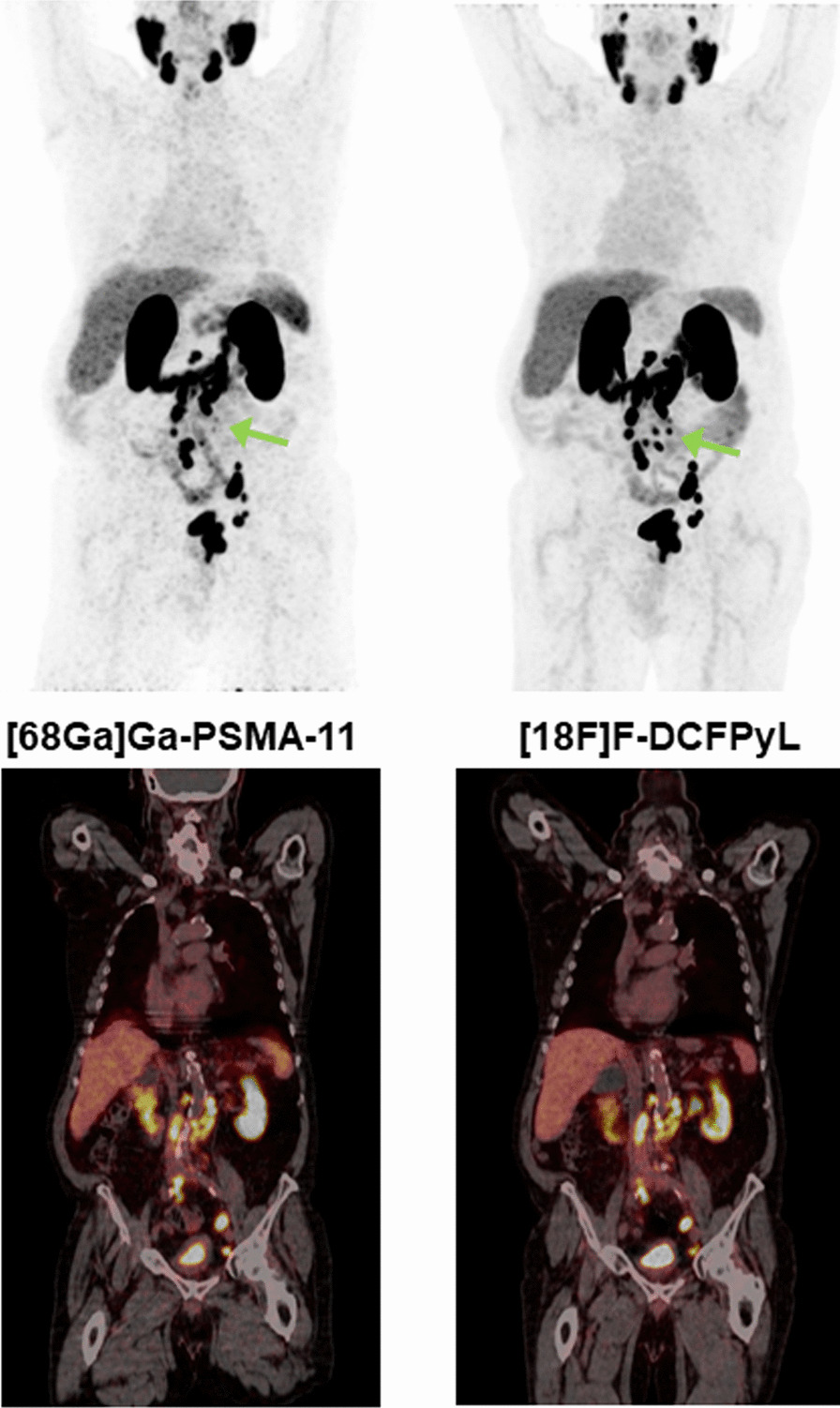
Image comparison of PET using [^68^Ga]Ga-PSMA-11 (left) and [^18^F]F-DCFPyL (right). PET images of patient no. 4 (lesions 6–12) are shown. Both PET with [^68^Ga]Ga-PSMA-11 and [^18^F]F-DCFPyL show evidence of local recurrence and extensive iliac and retroperitoneal lymph node metastases, as shown in the maximum intensity projection images (top row). The coronary fusion images (bottom row) show the bi-iliac and retroperitoneal lymph node metastases as expected, while the local recurrence is outside the imaged plane. Visual impression of tracer distribution confirms the statistically suspected comparability of tumour-to-liver ratios and tumour-to-mediastinum ratios between PET using [^68^Ga]Ga-PSMA-11 and [^18^F]F-DCFPyL. Furthermore, the well-known strengths of ^18^F-labelled PSMA tracers are apparent in the noticeable reduction in background noise and superior detectability of smaller lesions (green arrow) in the images from [^18^F]F-DCFPyL PET

**Fig. 2:**
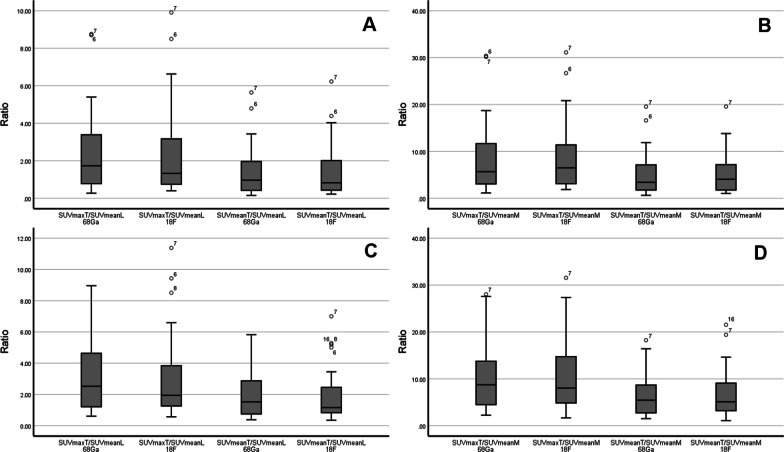
Boxplots of tumour-to-liver ratios and tumour-to-mediastinum ratios in [^68^Ga]Ga-PSMA-11 PET and [^18^F]F-DCFPyL PET. The top row (A + B) is based on OSEM and the bottom row (C + D) is based on HD/UHD reconstructed PET data. Tumour-to-liver ratios (A + C) and tumour-to-mediastinum ratios (B + D) were calculated using either the maximum or mean SUV of the tumour lesion or the mean SUV of the background region. Boxplots depict minimum, first quartile, median, third quartile and maximum as well as outliers (circles, number equals lesion ID). In general, these boxplots show that the different tumour-to-liver ratios and tumour-to-mediastinum ratios are comparable for [^68^Ga]Ga-PSMA-11 PET and [^18^F]F-DCFPyL PET. Wilcoxon matched-pair signed-rank test found no significant differences for OSEM as well as for HD/UHD-based ratios. OSEM ordered subset expectation maximisation, HD high definition, UHD ultra-high definition, PET positron emission tomography, SUV standardised uptake values corrected for body weight, SUV_maxT_/SUV_meanL_ Ratio of the maximum SUV in the tumour lesion to the mean SUV in the liver, SUV_meanT_/SUV_meanL_ Ratio of the mean SUV in the tumour lesion to the mean SUV in the liver, SUV_maxT_/SUV_meanM_ Ratio of the maximum SUV in the tumour lesion to the mean SUV in the mediastinum, SUV_meanT_/SUV_meanM_ Ratio of the mean SUV in the tumour lesion to the mean SUV in the mediastinum, ^68^Ga [^68^Ga]Ga-PSMA-11, ^18^F [^18^F]F-DCFPyL

A Wilcoxon matched-pair signed-rank test revealed no significant differences between [^68^Ga]Ga-PSMA-11-PET and [^18^F]F-DCFPyL-PET regarding tumour-to-liver ratios using either the maximum or the mean SUV of the tumour lesion for ratio calculation. This was demonstrated for data from both OSEM (SUV_maxT_/SUV_meanL_ p = 0.440, SUV_meanT_/SUV_meanL_ p = 0.989) and HD/UHD (SUV_maxT_/SUV_meanL_ p = 0.484, SUV_meanT_/SUV_meanL_ p = 0.346) reconstructed PET images. Median SUV_maxT_/SUV_meanL_ was 1.7 (0.3–8.8) in [^68^Ga]Ga-PSMA-11-PET vs. 1.3 (0.4–9.9) in [^18^F]F-DCFPyL-PET using OSEM and 2.5 (0.6–9.0) on [^68^Ga]Ga-PSMA-11-PET vs. 2.0 (0.6–11.4) on [^18^F]F-DCFPyL-PET using HD/UHD reconstruction methods. Median SUV_meanT_/SUV_meanL_ was 1.0 (0.2–5.6) on [^68^Ga]Ga-PSMA-11-PET vs. 0.8 (0.2–6.2) in [^18^F]F-DCFPyL-PET using OSEM and 1.5 (0.4–5.8) on [^68^Ga]Ga-PSMA-11-PET vs. 1.2 (0.4–7.0) in [^18^F]F-DCFPyL-PET using HD/UHD reconstruction methods. As an example, Fig. 1 shows PET images of patient no. 4 (lesions 6–12). Boxplots visualising tumour-to-liver ratios are shown in Fig. 2.

Furthermore, a Wilcoxon matched-pair signed-rank test revealed no significant differences between [^68^Ga]Ga-PSMA-11-PET and [^18^F]F-DCFPyL-PET regarding tumour-to-mediastinum ratios using either the maximum or the mean SUV of the tumour lesion for ratio calculation. This was demonstrated for data from both OSEM (SUV_maxT_/SUV_meanM_ p = 0.797, SUV_meanT_/SUV_meanM_ p = 0.764) and HD/UHD (SUV_maxT_/SUV_meanM_ p = 0.391, SUV_meanT_/SUV_meanM_ p = 0.278) reconstructed PET images. Median SUV_maxT_/SUV_meanM_ was 5.7 (1.1–30.4) on [^68^Ga]Ga-PSMA-11-PET vs. 6.5 (1.9–31.1) on [^18^F]F-DCFPyL-PET using OSEM, and 8.7 (2.2–28.0) in [^68^Ga]Ga-PSMA-11-PET vs. 8.0 (1.7–31.6) in [^18^F]F-DCFPyL-PET using HD/UHD reconstruction methods. Median SUV_meanT_/SUV_meanM_ was 3.4 (0.6–19.6) on [^68^Ga]Ga-PSMA-11-PET vs. 4.1 (1.0–19.6) on [^18^F]F-DCFPyL-PET using OSEM, and 5.5 (1.5–18.2) on [^68^Ga]Ga-PSMA-11-PET vs. 5.1 (1.1–21.5) on [^18^F]F-DCFPyL-PET using HD/UHD reconstruction methods. Boxplots visualising tumour-to-mediastinum ratios are shown in Fig. 2.

### *Contrast-to-noise ratios differ significantly between [*^*68*^*Ga]Ga-PSMA-11 and [*^*18*^*F]F-DCFPyL*

We calculated the contrast-to-noise ratio for each tumour lesion on [^68^Ga]Ga-PSMA-11-PET and [^18^F]F-DCFPyL-PET using OSEM as well as HD/UHD reconstruction algorithms. Contrast-to-noise ratios are shown in Tables 2 and 3.

**Fig. 3:**
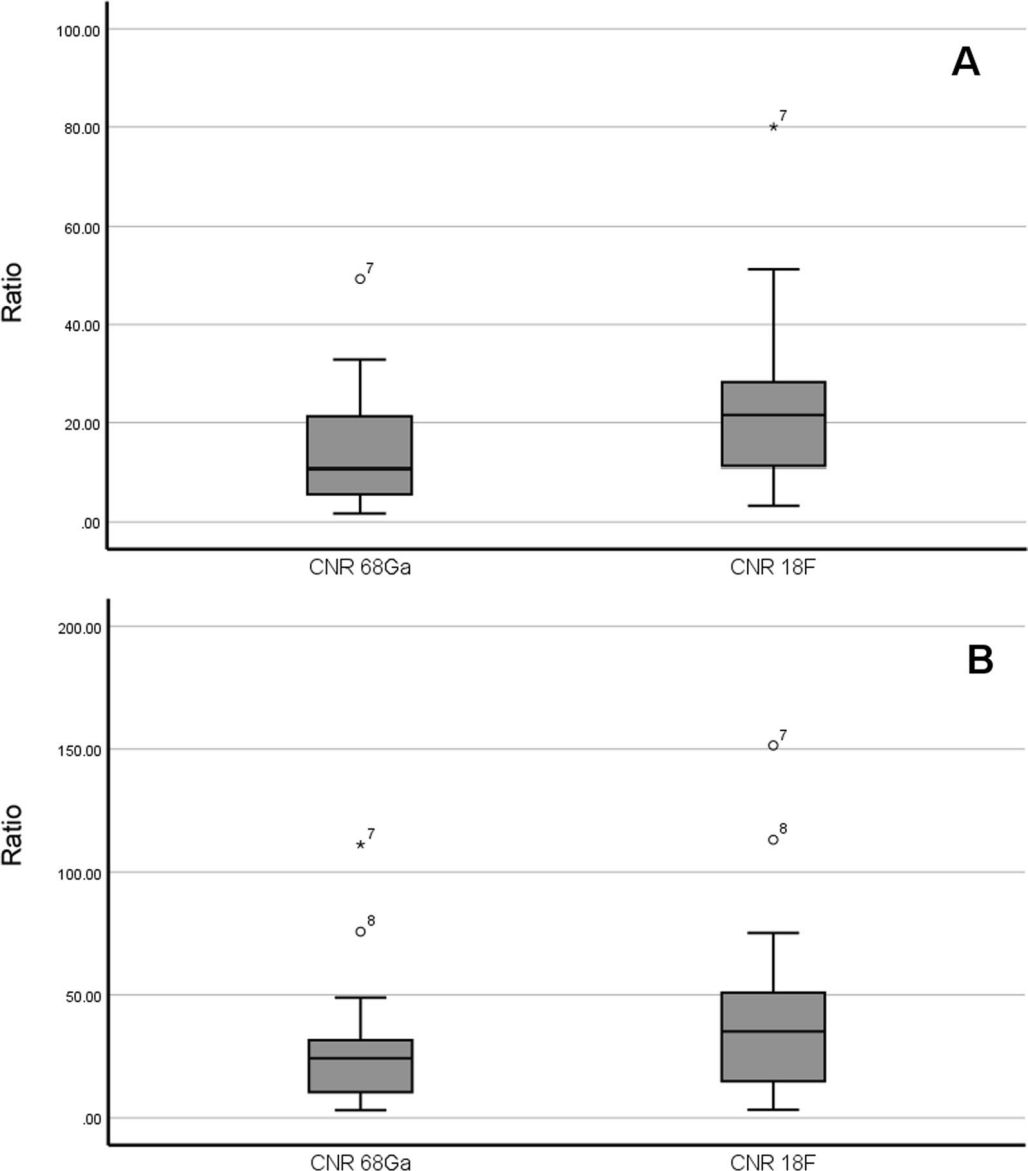
Boxplots of contrast-to-noise ratios in (**A**) OSEM and (**B**) HD/UHD reconstructed [^68^Ga]Ga-PSMA-11-PET and [^18^F]F-DCFPyL-PET. Boxplots depict minimum, first quartile, median, third quartile and maximum as well as outliers (circles, stars, number equals lesion ID). In general, these boxplots show that contrast-to-noise ratios were higher on PET using [^18^F]F-DCFPyL than on imaging with [^68^Ga]Ga-PSMA-11. Median contrast-to-noise ratios were 21.6 (3.3–80.0) in [^18^F]F-DCFPyL PET vs. 10.7 (1.8–49.3) in [^68^Ga]Ga-PSMA-11 PET using OSEM and 35.1 (3.3–151.5) in [^18^F]F-DCFPyL PET vs. 24.3 (3.1–111.2) in [^68^Ga]Ga-PSMA-11 PET using HD/UHD reconstruction methods. Wilcoxon test showed a highly significant difference regarding contrast-to-noise ratios in [^68^Ga]Ga-PSMA-11 PET and [^18^F]F-DCFPyL PET for both OSEM (p < 0.001) and HD/UHD (p < 0.001) reconstructed PET images. CNR contrast-to-noise ratio, ^68^Ga [^68^Ga]Ga-PSMA-11, ^18^F [^18^F]F-DCFPyL, OSEM ordered subset expectation maximisation, HD high definition, UHD ultra-high definition, PET positron emission tomography

A Wilcoxon matched-pair signed-rank test revealed a highly significant difference between [^68^Ga]Ga-PSMA-11-PET and [^18^F]F-DCFPyL-PET regarding contrast-to-noise ratios using both OSEM (p < 0.001) and HD/UHD (p < 0.001) reconstructed PET images. Overall, contrast-to-noise ratios were higher on [^18^F]F-DCFPyL-PET compared to those on [^68^Ga]Ga-PSMA-11-PET. Median contrast-to-noise ratio was 10.7 (1.8–49.3) on [^68^Ga]Ga-PSMA-11-PET vs. 21.6 (3.3–80.0) on [^18^F]F-DCFPyL-PET using OSEM, and 24.3 (3.1–111.2) on [^68^Ga]Ga-PSMA-11-PET vs. 35.1 (3.3–151.5) on [^18^F]F-DCFPyL-PET using HD/UHD reconstruction methods. Boxplots visualising contrast-to-noise ratios are presented in Fig. 3.

## Discussion

The following findings emerge from our analysis:

- A highly significant difference was observed between [^68^Ga]Ga-PSMA-11-PET and [^18^F]F-DCFPyL-PET regarding contrast-to-noise ratios. Overall, contrast-to-noise ratios were higher on [^18^F]F-DCFPyL-PET compared to those on [^68^Ga]Ga-PSMA-11-PET.
- No significant differences were observed between [^68^Ga]Ga-PSMA-11-PET and [^18^F]F-DCFPyL-PET regarding tumour-to-liver ratios or tumour-to-mediastinum ratios using either the maximum or the mean SUV of the tumour lesion for ratio calculation.
- All the above-mentioned findings could be demonstrated for PET data obtained using both OSEM and HD/UHD reconstruction methods.

Our first observation was that contrast-to-noise ratios were significantly higher on [^18^F]F-DCFPyL-PET than on [^68^Ga]Ga-PSMA-11-PET, demonstrating the better image quality with superior lesion detectability obtainable with [^18^F]F-DCFPyL-PET. This is consistent with previous observations of an improved image quality with ^18^F-labelled PSMA tracers. For example, data from our previous investigations suggest that ^18^F-labelled PSMA ligands are at least non-inferior to ^68^Ga-labelled PSMA ligands in detecting tumour lesions and offer some further advantages in detecting smaller tumour lesions at low PSA levels [20, 21, 22]. Higher contrast-to-noise ratios on [^18^F]F-DCFPyL-PET could be explained by the ability of ^18^F-labelled radiopharmaceuticals to be used for later imaging (due to a longer half-life and availability of higher activity provided by cyclotron production compared to ^68^Ga-labelled radiopharmaceuticals) [4].

Our second observation was that comparable tumour-to-liver ratios and tumour-to-mediastinum ratios were obtained with [^68^Ga]Ga-PSMA-11-PET and [^18^F]F-DCFPyL-PET. This is of particular interest, since PSMA-positive disease is a prerequisite prior to radioligand therapy with ^177^Lu-labelled PSMA ligands for patients with metastatic castration-resistant prostate cancer [3, 4, 10]. So far, in the approach taken by the pivotal PSMA-VISION trial, PSMA-positivity has been defined as tumoural PSMA expression above the liver background level on PET with [^68^Ga]Ga-PSMA-11 [3]. Despite their known advantages in image quality and logistics, ^18^F-labelled PSMA ligands were not considered in the PSMA-VISION trial [4, 23]. However, given their advantages, ^18^F-labelled PSMA ligands could become the future tracers of choice in PET diagnostics, as ^18^F-labelling make on-site radiolabelling unnecessary and would enable wider commercial distribution [4]. In this context, our present data suggest that PET with [^18^F]F-DCFPyL also offers the possibility of identifying PSMA-positive disease prior to radioligand therapy using the same qualitative liver-dependent approach as that proposed in the PSMA-VISION trial with [^68^Ga]Ga-PSMA-11 [3]. Given the comparable liver-to-background ratios obtained for [^68^Ga]Ga-PSMA-11 and [^18^F]F-DCFPyL, it is reasonable to consider tumour uptake above the liver background level as a definition of PSMA-positivity prior to radioligand therapy when using [^18^F]F-DCFPyL. This is consistent with the conclusions of Ferreira et al., who compared 34 patients who had received both [^68^Ga]Ga-PSMA-11-PET and [^18^F]F-DCFPyL-PET [24]. They showed an acceptable intra-individual agreement in terms of uptake in the liver background and took this to indicate a comparability of the two tracers in defining PSMA-positive disease prior to radioligand therapy [24]. However, in this previous study, no direct comparison of individual tumour-to-background ratios for different tracers in the same patients was performed [24]. Furthermore, our results support the consensus statement on the role of PSMA-PET published by the European Association of Nuclear Medicine and the European Association of Urology [25]. The participating experts agreed that the tracers studied here are equivalent to select patients for radioligand therapy [25]. This consensus is now underlined by direct evidence from our data.

As various ^18^F-labelled PSMA tracers are used in clinical evaluation, it is reasonable to question whether these tracers provide the same results in terms of predicting the likely success of radioligand therapy. In our view, it is quite possible that PSMA tracers such as [^18^F]F-PSMA-1007, which are predominantly eliminated via the liver, produce different results to those of their predominantly renally excreted competitors [26]. Thus in our opinion, it cannot be easily assumed that the qualitative liver-dependent approach proposed by the PSMA-VISION trial for [^68^Ga]Ga-PSMA-11 is equally suitable for tracers with distinctly different biodistribution [3]. However, it also remains unclear whether our results for [^18^F]F-DCFPyL can be extrapolated to other predominantly renally excreted tracers such as [^18^F]F-JK-PSMA-7 [26].

Our present study has some limitations. First, our comparison of [^68^Ga]Ga-PSMA-11 and [^18^F]F-DCFPyL was not designed as a prospective clinical trial. Second, our observations were focused on a highly select group of 11 patients with biochemical recurrence of prostate cancer due to the rare occurrence of dual PET protocol in clinical practice. Therefore, it must be taken into account that the patients studied were in an early stage of disease and thus should not receive radionuclide therapy according to current guidelines. It remains unclear whether our results can be transferred to patients with advanced disease who are eligible for radionuclide therapy, as changes in tumour biology or therapy-related effects may alter the uptake ratios. In addition, our retrospective data do not provide sufficient information on histopathological confirmation of suspicious PET findings. In order to obtain more solid evidence, it is necessary to validate our findings in a prospective trial with an appropriate study protocol and a larger number of patients. However, the option for a direct comparison within the same patients may represent a strength of the current study despite the small sample size. It will be generally difficult to obtain dual PET data for direct comparison and most previous studies have used matched-pair analyses to compensate for this lack of data [27]. It appears reasonable to assume that a reliable impression on the general tracer distribution behaviour with regard to tumour-to-background ratios can be drawn from the current sample. Therefore, despite its small cohort size, this study does help to provide further evidence of the comparability of these tracers.

## Conclusion

Our data showed that [^18^F]F-DCFPyl-PET and [^68^Ga]Ga-PSMA-11-PET provide comparable tumour-to-liver and tumour-to-mediastinum ratios. Therefore, a tumour uptake of [^18^F]F-DCFPyL above the liver background, like using [^68^Ga]Ga-PSMA-11, can be considered as equally suitable for defining PSMA-positivity by a semiquantitative assessment based on the liver background, e.g. prior to radioligand therapy with ^177^Lu-labelled PSMA ligands. In addition, our data suggest a tending advantage of [^18^F]F-DCFPyL in terms of lesion detectability.

## Data Availability

The datasets generated and analysed during this study are available from the corresponding author on reasonable request.

## Abbreviations

CT: Computed tomography
HD: High definition
OSEM: Ordered subset expectation maximisation
PET: Positron emission tomography
PSA: Prostate-specific antigen
PSMA: Prostate-specific membrane antigen
SD_N_: Standard deviation of SUV_meanN_
SUV: Standardised uptake value corrected for body weight
SUV_maxT_: Maximum SUV of the tumour lesion
SUV_meanL_: Mean SUV of the liver (hepatic background)
SUV_meanM_: Mean SUV of the mediastinum (mediastinal background)
SUV_meanN_: Mean SUV in the local (tumour surrounding) background
SUV_meanT_: Mean SUV of the tumour lesion
UHD: Ultra-high definition
VOI: Volume of interest
